# Schizotypy and Performance on an Insight Problem-Solving Task: The Contribution of Persecutory Ideation

**DOI:** 10.3389/fpsyg.2018.00708

**Published:** 2018-05-16

**Authors:** Jan Cosgrave, Ross Haines, Stuart Golodetz, Gordon Claridge, Katharina Wulff, Dalena van Heugten – van der Kloet

**Affiliations:** ^1^Department of Psychiatry, Perelman School of Medicine, University of Pennsylvania, Philadelphia, PA, United States; ^2^Sleep and Circadian Neuroscience Institute, Nuffield Department of Clinical Neurosciences, Medical Sciences Division, The Sir William Dunn School of Pathology, University of Oxford, Oxford, United Kingdom; ^3^Department of Statistics, Mathematical, Physical, and Life Sciences Division, University of Oxford, Oxford, United Kingdom; ^4^Oxford Smart Specs Group, Nuffield Department of Clinical Neurosciences, University of Oxford, Oxford, United Kingdom; ^5^Department of Experimental Psychology, Medical Sciences Division, University of Oxford, Oxford, United Kingdom; ^6^Social Work and Public Health, Department of Psychology, Faculty of Health and Life Sciences, Oxford Brookes University, Oxford, United Kingdom

**Keywords:** schizotypy, insight problem solving, paranoia, persecutory ideas, number reduction task, creativity

## Abstract

Insight problem solving is thought to underpin creative thought as it incorporates both divergent (generating multiple ideas and solutions) and convergent (arriving at the optimal solution) thinking approaches. The current literature on schizotypy and creativity is mixed and requires clarification. An alternate approach was employed by designing an exploratory web-based study using only correlates of schizotypal traits (paranoia, dissociation, cognitive failures, fantasy proneness, and unusual sleep experiences) and examining which (if any) predicted optimal performance on an insight problem-solving task. One hundred and twenty-one participants were recruited online from the general population and completed the number reduction task. The discovery of the hidden rule (HR) was used as a measure of insight. Multivariate logistic regression analyses highlighted persecutory ideation to best predict the discovery of the HR (OR = 1.05; 95% CI 1.01–1.10, *p* = 0.017), with a one-point increase in persecutory ideas corresponding to the participant being 5% more likely to discover the HR. This result suggests that persecutory ideation, above other schizotypy correlates, may be involved in insight problem solving.

## Introduction

The term schizotypy was first introduced in excess of 60 years ago to define a multi-dimensional personality type which encompasses a broad range of schizophrenia-like phenotypes and impairment (Kwapil and Chun, 2015). Schizotypal traits are commonly thought to lie upon a spectrum from healthy at one end, through to eccentricity and varied combinations of schizotypal traits, to florid psychosis at the other (Johns and van Os, 2001).

An interesting contrast in the schizotypy literature is that schizotypal traits have been shown to be associated with both cognitive impairments (Karagiannopoulou et al., 2016) and creative thinking (Holt, 2015). While sounding conflicting, the ability to perceive associations between seemingly disparate concepts is a valuable asset in creative problem solving but is also thought to underpin the development of the anomalous experiences observed in schizophrenia (Westerhausen et al., 2011).

The cognitive processes underlying creative thinking are thought to involve both divergent (DT; the relaxing of boundaries between concepts, which consequently allows for a broader associative network to develop; Guilford, 1956) and convergent thinking (CT; logically assembling connections and moving toward a single correct solution; Claridge and McDonald, 2009) abilities. The combination of these two parameters in creative thinking has often been examined through insight problem solving (DeYoung et al., 2008). Insight problem-solving tasks are intended to be simple (but not straightforward) and require conceiving of a diverse number of approaches to solving the problem (DT) and then subsequently filtering down these approaches to find the correct solution (Webb et al., 2017).

The current literature on schizotypy and creative thinking is mixed with few studies examining dimensional differences, limited sample sizes, and a disparity in how creativity is measured without due attention paid to the differences in problem-solving tasks (Webb et al., 2017). Caparrós et al. (2008) highlight that there is no clear or distinct pattern of association between cognitive abilities and schizotypy. Again, this may point to the importance of individual traits or dimensional differences in schizotypy.

As such, we wished to take an alternate approach to examining the relationship between schizotypal traits and creative thinking. As an exploratory study, our goal was to examine the ability of selected individual correlates of schizotypy [paranoid thinking, dissociation, unusual sleep experiences (including nightmares), and fantasy proneness] to predict the performance in an insight problem-solving task (which relies on both DT and CT). This approach is designed to examine the claim that the disparity observed in results from prior studies is in part driven by a correlate of schizotypy which may present only in certain subgroups of schizotypes (Kwapil and Chun, 2015).

Our selection of correlates was premised upon the work of Koffel and Watson (2009). Koffel and Watson (2009) proposed that unusual sleep experiences (with a specific focus on nightmares), dissociative experiences, and schizotypy all belong to a common domain and are more related to each other than other daytime symptoms (e.g., depression, anxiety, and substance abuse) and other sleep disturbances (e.g., insomnia). Further to this, previous work by Giesbrecht et al. (2007) and Sánchez-Bernardos and Avia (2006) found cognitive failures and creative experiences to be a strong correlate of cognitive disorganization and positive experiences dimensions of schizotypy. Finally, paranoia was also included as it is reported to be highly correlated with schizotypal traits but is not routinely examined in all schizotypal questionnaires (Kwapil and Chun, 2015).

**FIGURE 1:**
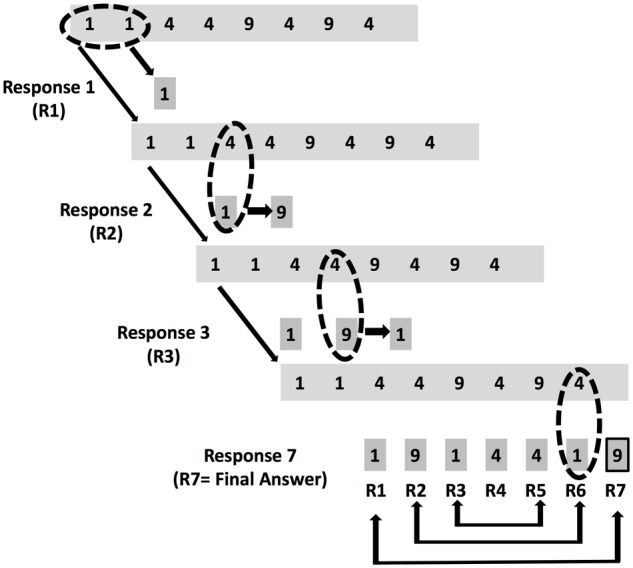
The number reduction task (NRT). This schematic diagram of the task is adapted from Verleger et al. (2013). Participants serially convert a given sequence composed of a total of 8 digits. Each sequence only contains the digits 1, 4, and 9. The objective is to convert this sequence into a new sequence in order to determine the final result of this trial (Response 7 [R7]). Unknown to the participants is the hidden structure of each sequence executed in every trial. As shown by the diagram, the digits used as last three responses mirror the previous three responses (illustrated by the pairwise arrows at the bottom of the figure). Consequently, the second response is always equal to the final response (i.e., R2 = R7; taken from Verleger et al., 2013).

We selected the number reduction task (NRT) as our insight problem-solving task (Haider and Rose, 2007). This task was chosen for two reasons. First, the NRT captures the core notions of DT and CT. Given the symmetry within the task (**Figure 1**), there are multiple ways of discovering a hidden rule (HR; DT) and seeing the problem beyond the two rules that every participant is presented with allows alternate ways to solve the problem (DT). Second, filtering through the possibilities to discovering “the HR” offers the optimal (and only) solution to the problem.

In sum, this is an exploratory study aiming to identify which correlates of schizotypy predict performance on an insight problem-solving task (as measured by the discovery of the HR in the NRT). Performance in the NRT has been found to be impacted by sleep (Verleger et al., 2013). To control for this, we set up two testing groups (a day and a night group). As such, we wished to explore which (if any) of our selected correlates of schizotypy predicted HR discovery accounting for the advantage of sleep.

## Materials and Methods

### Participants

One hundred and twenty-one participants (80 women; 40 men; one prefer not to say) were recruited via local email and poster advertisements located around Oxford. Their mean age was 23.4 years (SD: 5.1 years). We excluded one participant whose age (51) was dramatically outside of the range of ages of the rest of the participants.

In order to control for differences in pre-existing IQ, participants were required to report their current level of education, whether they were studying toward a higher level of education, and their subject area. Inclusion criteria entailed an age of 18 years and older, and proficiency in the English language. Exclusion criteria included pregnancy, medication use, history of brain injury, and travel through time zones within 2 weeks before the study.

### The Number Reduction Task (NRT; Haider and Rose, 2007)

The NRT was employed for our insight problem-solving task in this protocol. **Figure 1** displays an example trial of the task. Participants were presented with a sequence of eight digits composed only of 1s, 4s, and 9s. These sequences varied from trial to trial. The final response of a given sequence had to be given before the participant could move onto the next trial. In order to achieve this, the participant serially processed two digits from each sequence using two simple rules to guide their responses. These rules were:

(1) The “same” rule: when comparing two identical digits, the response is the same digit (e.g., 1 and 1 gives 1; R1 in **Figure 1**).
(2) The “different” rule: when comparing two different digits in the sequence, the response is the remaining third digit (e.g., 1 and 4 gives 9; R2 in **Figure 1**).

Unknown to the participants was that each sequence could be computed using a shortcut known as the “HR.” Within each sequence, the responses mirrored each other and importantly, the second response (R2) always mirrored the final response (R7). Discovery of this hidden symmetry within each sequence allowed the participant to compute the final response using R2, bypassing the computation of R3–R7 and thus radically reducing their reaction time for each sequence.

Thus, the NRT is an algorithmic task which measures reaction time, and, as such, participants were asked to complete the task as quickly and correctly as possible. We developed an online version of the NRT, which participants completed from their own homes. Previous studies have shown that sleep can influence the likelihood of discovering the HR within the NRT (Wagner et al., 2004; Verleger et al., 2013). To account for this, participants were randomly allocated to either a day or night group. The task included 15 practice trials for which a minimum score of 12 correct responses was needed to gain access to the first session. To help promote understanding of the task, participants could jot down each of their responses within each trial and were able to see the correct response to each of the algorithms during the practice session. They were allowed to repeat the practice round as many times as they wished.

**FIGURE 2:**
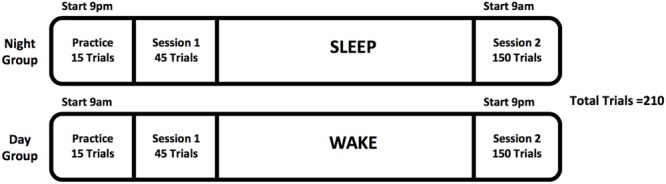
Study protocol.

Participants then progressed to Session 1 of the main task, comprising 45 trials. The task was programmed to allow access only at the specific times of the experiment (either 9 a.m. or 9 p.m.). After an 11-h rest period (either with or without a bout of sleep), the participant then completed Session 2, composed of 150 trials. The webpage for completing the task was timed to open after 11 h and remained open for a total of 2 h. Attempts to access the experiment outside of these times resulted in an error message. Discovery of the HR was defined as having three consecutively correct final responses all below 2 s in reaction time during Session 2. For a diagrammatic version of the protocol, please see **Figure 2**.

### Assessment of State and Trait Correlates of Schizotypy, Sleep, and Chronotype

Participants completed a battery of primary measures, including the Dissociative Experiences Scale-II (DES-II; Bernstein-Carlson and Putnam, 1993), the Green Paranoid Thoughts Scale (GPTS; Green et al., 2008), the Creative Experiences Questionnaire (CEQ; measuring fantasy proneness; Merckelbach et al., 2001), the Cognitive Failures Questionnaire (CFQ; Broadbent et al., 1982), the Iowa Sleep Experiences Survey (Watson, 2001), the Nightmare Frequency Questionnaire (Belicki, 1992), and the Nightmare Distress Questionnare (Martínez et al., 2005). The nightmare measures were added as they have been specifically highlighted within unusual sleep experiences as being important to schizotypal traits, so much so they have been shown to have stronger relationships to schizotypy and dissociation than those seen with anxiety and depression and schizotypy and dissociation (Koffel and Watson, 2009). As control measures, we added the State-Trait Anxiety Inventory (Spielberger et al., 1970; Marteau and Bekker, 1992) and Beck’s Depression Inventory (Beck et al., 1961) to ensure that our results could not be explained through negative affect. Finally, we added the Morning–Eveningness Questionnaire (MEQ; Horne and Ostberg, 1975) to account for the fact that the participant’s performance may be optimized by their circadian preference aligning with the timing of the opening session: an example would be an evening type starting the task at 9 p.m. The internal consistency for each measure was calculated from the current study using Cronbach’s α.

### Primary Measures

#### Green Paranoid Thoughts Scale (GPTS; Cronbach’s α = 0.93; Green et al., 2008)

The GPTS is a trait measure of paranoia. It contains 32 items which can be divided into two 16-item subscales: social reference and persecutory thinking. All items are rated on a five-point scale, with higher scores indicating greater levels of paranoid thinking. It has good internal consistency and test–retest reliability (Green et al., 2008).

#### Dissociative Experiences Scale-II (DES; Cronbach’s α = 0.90)

The DES-II (Bernstein-Carlson and Putnam, 1993) is a self-report scale of trait dissociation. It requires participants to indicate on 100 mm visual analog scales (anchors: 0 = never; 100 = always) to what extent they experience 28 dissociative experiences in daily life. Van Ijzendoorn and Schuengel (1996) provide meta-analytic evidence for the sound psychometric properties of the DES.

#### Creative Experiences Questionnaire (CEQ; Cronbach’s α = 0.80; Merckelbach et al., 2001)

The CEQ is an instrument to measure fantasy proneness. It consists of 25 yes/no items measuring daydreaming, intense fantasies, and imagination. A total CEQ score is obtained by taking the sum of the number of items that are endorsed.

#### Cognitive Failures Questionnaire (CFQ; Cronbach’s α = 0.91)

The CFQ (Broadbent et al., 1982) is a 25-item self-report instrument assessing everyday lapses in perception, attention, and actions. Participants are requested to indicate on five-point scales how often they experienced each cognitive failure during the past month (anchors: 0 = never; 4 = very often). These scores are added together to obtain a total CFQ score, with higher scores indicating a higher frequency of self-reported failures.

#### Iowa Sleep Experiences Survey (ISES; Cronbach’s α = 0.90)

The ISES (Watson, 2001) consists of 18 questions, which assess the frequency of various sleep- and dream-related experiences, which are rated on a seven-point scale (anchors: 1 = never; 7 = several times a week). The ISES consists of two separate subscales that measure general sleep experiences and lucid dreaming. It has an acceptable internal consistency (coefficient α = 0.85; Watson, 2001).

#### Nightmare Frequency Questionnaire (NFQ; Cronbach’s α = 0.60; Belicki, 1992)

The NFQ assesses nightmare frequency as a continuous variable. Both “nights with nightmares” per unit of time (e.g., per week and per month) and actual “number of nightmares” are determined. Test–retest reliability on the NFQ yielded both correlation coefficients and weighted kappas greater than 0.85 in all analyses (Krakow et al., 2002).

#### Nightmare Distress Questionnaire (NDQ; Cronbach’s α = 0.99; Martínez et al., 2005)

The Nightmare Distress Questionnaire (NDQ) includes 13 items rated on a five-point scale to assess the degree of distress attributed to nightmares by nightmare sufferers. The NDQ shows acceptable psychometic properties with good validity and reliability (Martínez et al., 2005).

### Control Measures

#### Beck’s Depression Inventory-II

The Beck Depression Inventory-II (BDI-II; Cronbach’s α = 0.89) is a 21-item self-report scale for the assessment of depression over the past fortnight. It is rated on a four-point scale (0–3), with the total score ranging from 0 to 63 points. Higher scores indicate higher depression (Beck et al., 1961).

#### State Trait Anxiety Inventory – Short Form Version (STAI; Cronbach’s α = 0.87; Spielberger et al., 1970; Marteau and Bekker, 1992)

The STAI is composed of six brief items to each of which there are four possible responses (“not at all,” “somewhat,” “moderately,” and “very much”). The scores across the six items are summed to provide the STAI score. It has acceptable reliability and produces scores similar to its full form (Marteau and Bekker, 1992).

#### Morning–Eveningness Questionnaire (MEQ; Cronbach’s α = 0.80)

The MEQ (Horne and Ostberg, 1975) is a 19-item measure designed to assess time-of-day preference. The MEQ is the most commonly used measure of time-of-day preference (Adan et al., 2012). Scores on the MEQ range from 16 points (strong evening preference) to 86 points (strong morning preference).

### Statistics

Statistical analyses were performed within the R statistical environment (R Core Team, 2012). Pearson product-moment correlations between baseline measures were calculated, with a Bonferroni correction used when assessing significance to adjust for multiple comparisons (see the legend of **Table 2** for details).

To more thoroughly assess the impact of these baseline measures on the attainment of the HR, we performed multivariate logistic regression modeling. These models were used to estimate the probability of attainment of the HR given a set of predictor variables (i.e., our baseline measures). Regression coefficients were transformed into odds ratios.

Given the large number of possible combinations of the baseline measures for inclusion in a model, we performed model selection based upon the Akaike information criterion (AIC) and Bayesian information criterion (BIC) to objectively provide a set of candidate models for further consideration. These measure the relative quality of a collection of models and feature a penalty for the model’s complexity, thus discouraging overfitting. This penalty is more stringent with the BIC than the AIC, thus favoring simpler models.

We considered the output from forward selection and backward elimination, which are standard model selection procedures. Beginning with a simple intercept-only model (with no predictor variables), forward selection iteratively adds the predictors offering maximal reduction to the information criterion used (AIC or BIC), until no further reduction is possible. Backward elimination instead iteratively removes predictor variables from a complex model, until no further reduction in AIC/BIC is possible.

## Results

Two hundred and nine participants registered for the experiment, of which 1.4% (*n* = 3) did not successfully get a score of 12 on the NRT and thus failed to progress to Session 1. A further 12.9% (*n* = 27) completed the first session but failed to either complete the questionnaires or the second session within the allocated 11-h time window; 2.4% (*n* = 5) needed to be excluded due to technical difficulties with the webpage when the participant was completing the task, 14.4% (*n* = 30) stopped responding to emails and were removed from the study, and 4.3% (*n* = 9) dropped out for other reasons (personal time constraints, etc.). Finally, 7.2% (*n* = 15) were excluded from the analysis as they failed to reach 50% accuracy in their responses, highlighting that they may have been hitting responses randomly, or did not fully understand the task. We further excluded one participant whose age (51) was dramatically outside of the range of ages of the rest of the participants.

All remaining participants (*n* = 120) had completed secondary school; 95 participants (78.5%) were currently studying toward further education. Of those not currently in education (21.5%), only one participant did not possess a bachelors degree. Thus, 99.2% of the participants were either currently completing a bachelor’s degree or had already completed one. A distribution of each of the subject categories and their respective HR attainment can be seen in **Table 1**. Mean scores, SDs, and Pearson product-moment correlations of each of the psychiatric measures are displayed in **Table 2**.

**Table 1:** Distribution of HR attainment across different subject areas.

Subject Area	No HR	HR	% of HR attainment
Medical sciences	19	10	34.5
Humanities	34	12	26.1
Math/physical science	18	7	23.0
Others	13	7	35.0

**Table 2:** Mean scores, SDs (in brackets), and Pearson product-moment correlations for baseline psychiatric measures (*N* = 120).

	Mean *(SD)*	BDI	CEQ	CFQ	DES	ISES	LUC	MEQ	NFQ	NDQ	STAI	GPT
BDI	6.93 (7.00)	–										
CEQ	7.61 (4.56)	0.35*	–									
CFQ	1.57 (0.57)	0.43*	0.38*	–								
DES	9.79 (7.84)	0.54*	0.52*	0.52*	–							
ISES	27.55 (14.43)	0.42*	0.55*	0.31*	0.49*	–						
LUC	4.79 (4.11)	0.16	0.35*	0.04	0.22	0.59*	–					
MEQ	45.55 (8.99)	–0.17	–0.04	–0.24	–0.07	–0.12	0.03	–				
NFQ	13.60 (12.53)	0.37*	0.42*	0.18	0.36*	0.77*	0.38*	–0.10	–			
NDQ	33.36 (5.04)	–0.42*	–0.36*	–0.32*	–0.37*	–0.52*	–0.18	0.12	–0.58*	–		
STAI	11.86 (3.90)	–0.72*	–0.26	–0.42*	–0.37*	–0.39*	–0.14	0.16	–0.38*	0.37*	–	
GPT(P)^a^	22.32 (9.35)	0.34*	0.21	0.13	0.41*	0.16	0.03	–0.11	0.11	–0.28	–0.27	–
GPT(SR)^a^	29.64 (10.40)	0.36*	0.28	0.43*	0.52*	0.28	–0.04	–0.11	0.20	–0.36*	–0.36*	0.70*

*BDI, Beck’s Depression Inventory; CEQ, Creative Experiences Questionnaire; CFQ, Cognitive Failures Questionnaire; DES, Dissociative Experiences Scale; ISES, IOWA Sleep Experiences Survey; LUC, Lucid Dreaming subscale of the ISES; MEQ, Morning–Eveningness Questionnaire; NFQ, Nightmare Frequency Questionnaire; NDQ, Nightmare Distress Questionnaire; STAI, State Trait Anxiety Inventory; GPT(P), Green Paranoid Thoughts (persecutory subscale); GPT(SR), Green Paranoid Thoughts (social reference subscale). A Bonferroni correction was applied to adjust for multiple comparisons, using a family-wise error rate (FWER) of α = 0.05.*

*^*a*^The GPT (P) and (SR) have only 108 compete cases. ^∗^*p* < α/*m* (where *m* = 66 is the number of statistical tests being performed).*

### Hidden Rule Attainment

Of 120 participants, 36 participants (30.0%) discovered the HR (i.e., had three consecutive correct final responses at a reaction time of 2 s or below). Four participants (three in the day group and one in the night group) attained the HR in their first session.

### Impact of Circadian Preference

Of the participants, 5.83% were definite evening types, 28.33% were moderate evening types, 57.5% were intermediate types, and 8.33% were moderate morning types. There were no extreme morning types in the sample. To control for circadian perferences on HR attainment we investigated whether starting the task at 9 p.m. for evening types helped promote insight.

Interestingly, 44% of the evening types (7 of 16 participants) attained the HR when starting at 9 p.m., as opposed to 30% (7 of 23 participants) of evening types who started at 9 a.m. This failed to reach significance using Fisher’s exact test for count data (*p* = 0.74). Similarly, only 17% (one of six participants) of morning types attained the HR when starting at 9 p.m. as opposed to 75% (three of four participants) who attained the HR when they started the task at 9 a.m. Significance could not be tested due to so few moderate morning types in the data set (*n* = 10).

### “Sleep Inspires Insight”

To control for the effect of sleep on the attainment of the HR, we analyzed differences in attainment of the HR between the day and night groups. We excluded the four participants who attained the HR in Session 1, which is in line with what has previously been done using the NRT (Wagner et al., 2004; Yordanova et al., 2008).

In both the day and the night groups, 16 out of 58 participants (27.6%) gained insight into the HR. Using Fisher’s exact test, we found no evidence for a significant difference in HR attainment between groups (*p* = 1.0). We therefore decided to collapse across groups for further analyses.

### Further Exploration Into the Hidden Rule

Possible predictor variables for inclusion in multivariate logistic regression models for HR attainment were BDI, ISES, lucid dreaming (a subscale of the ISES), DES, MEQ, NFQ, NDQ, STAI, CEQ, CFQ, and paranoia. Paranoia was split into the two subscales of the GPT: persecutory ideas (PER) and social reference (SR). Gender, age, educational level, and subject area were also considered as predictor variables to control for their impact (if any) on HR attainment.

When using AIC, the backward elimination process resulted in a model with only the PER score as a predictor variable. The forward selection process led to a more complex model, also featuring the PER score, but with the BDI and DES scores as well. When using BIC, both backward elimination and forward selection resulted in the model with only the PER score as a predictor variable.

**Table 3** provides a summary of our candidate models: the baseline intercept-only model (Model 0), the two models described above (Models 1 and 3, respectively), and models with PER and one of BDI and DES as predictors (Models 2a and 2b).

**Table 3:** Summary of multivariate logistic regression on hidden rule attainment (*N* = 108).

	Variable	β (SE)	Wald	*p*	AIC	BIC	χ^2^ (df)	*p*
Model 0	*intercept*	–0.74 (0.21)	–3.58	0.0004^∗∗^				
					**138.06**	**140.74**		
Model 1	*intercept*	–1.91 (0.55)	–3.45	0.0006^∗∗^				
	GPT (PER)	0.05(0.02)	2.32	0.021^∗^				
					**134.32**	**139.69**	**5.74 (1)**	**0.017^∗^**
Model 2a	*intercept*	–1.88 (0.56)	–3.34	0.0009^∗∗^				
	GPT (PER)	0.07 (0.03)	2.72	0.007^∗^				
	BDI	–0.06 (0.04)	–1.66	0.096				
					**133.15**	**141.20**	**3.17 (1)**	**0.075**
Model 2b	*intercept*	–1.96 (0.56)	–3.49	0.0005^∗∗^				
	GPT (PER)	0.04 (0.02)	1.85	0.064				
	DES	0.20 (0.28)	0.70	0.48				
					**135.84**	**141.20**	**0.48 (1)**	**0.49**
Model 3	*intercept*	–1.98 (0.58)	–3.43	0.0006^∗∗^				
	GPT (PER)	0.06 (0.03)	2.23	0.026^∗^				
	BDI	–0.10 (0.05)	–2.16	0.031^∗^				
	DES	0.58 (0.34)	1.69	0.091				
					**132.22**	**142.95**		

*BDI, Beck’s Depression Inventory; DES, Dissociative Experiences Scale; GPT (PER), Green Paranoid Thoughts (Persecutory subscale); AIC, Akaike information criterion; BIC, Bayesian information criterion; df, degrees of freedom. ^∗^*p* < 0.05 and ^∗∗^*p* < 0.001.*

Although the most complex of these models (Model 3) has the lowest AIC, it also has the highest BIC, and we note that there is a strong correlation between DES and BDI (*r* = 0.538). The model with only PER (Model 1) is the only model with a lower BIC than the baseline model (Model 0), so we proceed to use this model to predict HR attainment.

Selecting between these models instead using a likelihood-ratio-based test would lead to the same conclusion. The addition of PER to the intercept-only model offered a significant improvement (*p* = 0.017); however, subsequently adding BDI or DES to the model did not (*p* = 0.075 and *p* = 0.49, respectively).

**FIGURE 3:**
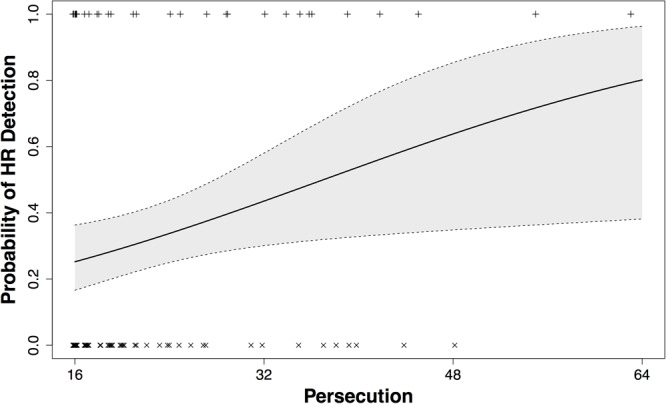
Probability of hidden rule detection as a function of persecutory ideation. The black line represents predictions from our model 1 for the probability of hidden rule attaining as a function of persecution. Persecution is measured using the persecutory ideas subscale on the GPTS (Green et al., 2008). Individuals with a persecution score of 16, the lowest possible score on this scale, show a relatively low probability of discovering the HR. However, as the persecutory ideation increases, we notice a steady increase in the probability of discovering the HR. This would suggest a relation between divergent thinking (as measured by hidden rule attainment) and persecutory ideas. The persecution scores of the participants who did not attain the hidden rule are plotted using crosses (x); similarly, the persecution scores for those who did are plotted using plusses (+). The shaded region around the predicted line represents the 95% confidence interval for the predicted hidden rule attainment probability. This widens as persecution increases, reflecting the limited number of participants with extremely high persecution scores.

Based upon the selected model (Model 1), the HR discovery rate increased 5% for every one-point increase reported in PER on the GPTS [OR = 1.05 (95% CI 1.01–1.10)]. This predicted increase in the probability of HR discovery is displayed in **Figure 3**.

## Discussion

In a group of 120 individuals from the general population recruited and tested online, analysis of schizotypy correlates, sleep, and insight (via discovery of the HR) in the NRT as an insight problem-solving task revealed two main findings. First, contrary to our hypothesis, the level of attainment of the HR was not higher for those who slept between test sessions. Second, the probability of discovering the HR increased with increasing persecutory ideation, which suggests that persecutory paranoia may relate to insight problem solving and hence creative thinking.

### “Sleep Inspires Insight?”

Based on preceding studies by Wagner et al. (2004) and Yordanova et al. (2008), we predicted that there would be a significantly higher attainment of the HR for those who slept, but this was not observed in the present study. There are several possible explanations for this discrepancy: (1) the use of an online at-home version resulting in a less controlled (but more naturalistic) environment; (2) participants were able to have the amount of sleep they wished without being monitored; (3) we had little information regarding activities in the 11-h period that could have potentially affected attainment of the HR; and (4) this effect is only observed when compared with sleep deprivation. A review by Verleger et al. (2013) investigates the role of sleep in the discovery of the HR with the NRT; however, to date, there is only one study which unambiguously tests whether sleep promotes insight (with a sleep deprivation and day comparison; Wagner et al., 2004). This study had more tightly controlled laboratory settings and a sleep deprivation group as part of the design. However, our study nearly doubled the sample size. Thus it could be that the HR attainment differs between laboratory and home environments, that differences in inclusion criteria negate the effect (the original study used only university students), or that with a larger sample size the outcome cannot be replicated.

### Subclinical Paranoia and Cognitive Performance

We found the PER score on the GPTS was a significant predictor for discovering the HR on the NRT over and above single or combined our selected schizotypy correlates. Thus, an interpretation of our results could be that subclinical paranoia is cognitively advantageous as it subserves cognitive processes involved in DT and CT. The research conducted on cognitive performance in subclinical paranoia is sparse to date. However, a study by Combs et al. (2003) did find that a high subclinical paranoia group outperformed a comparatively low subclinical paranoia group on both social and non-social stimuli in an implicit learning task. The high paranoia group was also more confident in their ratings relative to the low group for all stimuli. However, considerably more research is needed to be able to be able to substantiate this relationship.

### The Presence of Paranoia in Schizophrenia

Another avenue to examine the relationship between cognitive performance and paranoia would be to explore comparisons between patients with schizophrenia who endorse paranoia compared to schizophrenia patients who do not. Growing evidence suggests that fundamental cognitive processing is likely to differ between paranoid and non-paranoid traits of schizophrenia. Bark et al. (2005) found schizophrenia patients who endorse paranoia to outperform schizophrenia patients who do not in the object alternation task (Freedman, 1990) and the Iowa gambling task (Bechara, 2007), while Lewis and Garver (1995) found patients who experience paranoia in the context of schizophrenia to outperform schizophrenia patients without paranoia in affect recognition abilities. Moreover, Huanzhong et al. (2010) found that these patterns were present in comparisons of children suffering from schizophrenia with paranoia compared to those without paranoia when using the clinical memory scale and Raven’s Matrices. Notably, patients suffering from schizophrenia spectrum disorders with current delusions have also been associated with “jumping to conclusions” (JTC) reasoning biases but at present it is too early to tell how this might relate to the findings of the current study (Garety et al., 2013).

Taken together, this could suggest that the presence of paranoia in schizophrenia, despite being severely emotionally debilitating, may confer a cognitive advantage when compared to patients without paranoia in schizophrenia and this pattern appears in both child and adult populations.

Why would experiences of paranoia display any advantage when it is related to debilitating mental health disorders like schizophrenia? Schizotypal traits are exemplary of traits known to be associated with illness but they are present and prevalent in the general population (Claridge and Blakey, 2009). Examples of this can be found in the relatives of patients suffering from schizophrenia who show similar but attenuated deficits (ex: Lawrie et al., 2001). Taken together, this provides evidence for a genetic continuum and it seems plausible that the evolutionary advantage would lie not with those who have the disorder but indeed with those who share specific traits or even genes (Nichols, 2009). Normativity in mental health research often relies on the assumption that subclinical traits or symptoms still lie within the realms of socially deviant behavior (Muders, 2014), our results in line with what has been proposed by Claridge and Blakey (2009) contest that idea.

A number of caveats merit mention. Compared to studies that have also employed the NRT (Verleger et al., 2013), we had little control over the parameters of sleep, as participants were able to have the amount of sleep they wished. Second, the NRT was completed in a home environment; this resulted in a less controlled setting. Third, the inclusion criteria for this study were broad. This combined with the fact that it was web-based study may provoke a number of uncontrolled biases in recruitment, subjects’ responses to the questionnaires and/or NRT performance.

To our knowledge, this is the first study to explore correlates of schizotypal traits an insight problem-solving task and highlighting the potential role for persecutory thoughts. The full significance of the prediction may become apparent in planned longitudinal studies investigating whether there is a neuro-anatomical difference, which may expose the potential mechanisms of paranoid thought in both clinical and subclinical populations.

## Ethics Statement

All subjects gave written informed consent in accordance with the Declaration of Helsinki. The protocol was approved by the Medical Sciences Inter Division Research Ethics Committee (MSD-IDREC-C1-2014-052) at the University of Oxford.

## Author Contributions

JC aided in conceptualization and design with DvHvdK, collaboratively undertook recruitment with DvHvdK, wrote the manuscript, edited subsequent versions, and collaboratively analyzed the data with RH. RH undertook the analyses for the manuscript as well as extensively editing the manuscript to bring it to its final version. SG developed the computer program and software that allowed the cognitive task to work online, also offered considerable advice on designing the protocol, and extensively edited the manuscript to its final version. GC provided considerable intellectual guidance based on his expertise in the area and edited the manuscript to its final version. KW again provided intellectual expertise and did extensive editing of the manuscript to bring it to its final version. DvHvdK conceptualized the study, conducted recruitment, provided the funding for the study, and provided the intellectual backbone for the design.

## Conflict of Interest Statement

The authors declare that the research was conducted in the absence of any commercial or financial relationships that could be construed as a potential conflict of interest.
